# *TNF-α* promoter hypomethylation is frequent in oncopediatric patients who recovered from mucositis

**DOI:** 10.1590/1807-3107bor-2024.vol38.0042

**Published:** 2024-05-13

**Authors:** José Maria Chagas VIANA FILHO, Marina de CASTRO COÊLHO, José Nunes de QUEIROZ NETO, Beatriz Fernandes de SOUZA, Ana Maria Gondim VALENÇA, Naila Francis Paulo de OLIVEIRA

**Affiliations:** (a) Universidade Federal da Paraíba – UFPB, Centro de Ciências da Saúde, Programa de Pós Graduação em Odontologia, João Pessoa, PB, Brasil.; (b) Universidade Federal da Paraíba – UFPB, Centro de Ciências Exatas e da Natureza, Departamento de Biologia Molecular, João Pessoa, PB, Brasil.

**Keywords:** Mucositis, DNA Methylation, Epigenomics, Tumor Necrosis Factor-alpha, Inflammation

## Abstract

The aim of this study was to investigate the DNA methylation profile in genes encoding catalase (*CAT*) and superoxide dismutase (*SOD3*) enzymes, which are involved in oxidative stress mechanisms, and in genes encoding pro-inflammatory cytokines interleukin-6 (*IL6*) and tumor necrosis factor-alpha (*TNF-α*) in the oral mucosa of oncopediatric patients treated with methotrexate (MTX^®^). This was a cross-sectional observational study and the population comprised healthy dental patients (n = 21) and those with hematological malignancies (n = 64) aged between 5 and 19 years. Oral conditions were evaluated using the Oral Assessment Guide and participants were divided into 4 groups: 1- healthy individuals; 2- oncopediatric patients without mucositis; 3- oncopediatric patients with mucositis; 4- oncopediatric patients who had recovered from mucositis. Methylation of DNA from oral mucosal cells was evaluated using the Methylation-Specific PCR technique (MSP). For *CAT*, the partially methylated profile was the most frequent and for *SOD3* and *IL6*, the hypermethylated profile was the most frequent, with no differences between groups. For *TNF-α*, the hypomethylated profile was more frequent in the group of patients who had recovered from mucositis. It was concluded that the methylation profiles of *CAT, SOD3*, and *IL6* are common profiles for oral cells of children and adolescents and have no association with oral mucositis or exposure to chemotherapy with MTX^®^. Hypomethylation of *TNF-α* is associated with oral mucosal recovery in oncopediatric patients who developed oral mucositis during chemotherapy.

## Introduction

Pediatric patients with hematological tumors receiving chemotherapy treatment with methotrexate (MTX^®^) are frequently affected by oral mucositis. This inflammation is characterized by an ulcerative lesion associated with pain, resulting in difficulty in eating, drinking and speaking, and requiring nutritional support (nasogastric tube) in cases of severe oral mucositis.^1,2^ It can worsen the patient’s cachexia, decrease their quality of life and lead to temporary interruption of chemotherapy treatment for the recovery of the oral mucosa, which negatively impacts the patient’s treatment.^1,3^

The pathobiology of oral mucositis has five phases that start with chemotherapy and end with healing. In the initiation phase (1), chemotherapy induces the formation of reactive oxygen species, causing cellular damage to the epithelium and subepithelial mucosa. In the message generation phase (2), a series of transcription factors are activated along with the production of pro-inflammatory cytokines, such as tumor necrosis factor alpha (TNF-α), interleukin-1 (IL-1), interleukin-6 (IL-6), and C-reactive protein (CRP). In the signal amplification phase (3), inflammatory modulators are activated and released into the interstitial space leading to edema. In the ulceration phase (4), cytotoxic agents reduce mitosis of dividing epithelial cells in the oral cavity causing atrophy and ulceration, exacerbating the pain, and impairing the patient’s life. Opportunistic microorganisms in the oral cavity rapidly colonize these areas, increasing the risk of superinfection. In the healing phase (5), epithelial cells begin to proliferate and differentiate, initiating the healing of the mucosal tissue.^4,5^ Two aspects are important for the development of mucositis, especially in phases 1, 2 and 3 (initial stages), namely: oxidative stress and the production of inflammatory cytokines.

Oxidative stress occurs when there is an imbalance between free radical (oxidants) formation and the ability of the antioxidant system to neutralize them.^6^ Regarding the relationship between oxidative stress and oral mucositis, oxidative stress has been proposed as a mediator of MTX^®^ toxicity. Ahmed et al.^7^ observed a reduction in the activity of the antioxidant enzymes superoxide dismutase (SOD) and catalase (CAT) in the oral mucosa of rats. The authors showed that MTX^®^ result in impairment of the endogenous antioxidant defense system (antioxidant enzymes), thus exposing cells to free radicals, indicating that MTX^®^ can evoke extensive tissue damage mediated by oxidative stress.

Phases 2 and 3 of oral mucositis are related to the activation and production of cytokines, and this seems to be mediated by oxidative stress.^5^ The contemporary pathobiological model includes activation of nuclear transcription factor (NF-κB), which in turn positively regulates up to 200 genes, including genes encoding pro-inflammatory cytokines such as tumor necrosis factor-alpha (TNF-α) and interleukin-6 (IL-6).^4,8^

The genes that encode the antioxidant enzymes catalase and superoxide dismutase and the pro-inflammatory cytokines tumor necrosis factor-alpha and interleukin-6, are regulated by epigenetic mechanisms and are associated with disease.^9-12^ Epigenetics is information that is “above the DNA”, which controls gene expression, and is both heritable and reversible. Epigenetic marks include DNA methylation, chemical changes in histones, and small non-coding RNAs. These marks are associated with several tumors and inflammatory and mental diseases and are modulated by extrinsic factors including drugs, being a target for these agents or a product of adverse reactions.^13^

DNA methylation is the most studied epigenetic marker and is associated with a complete decrease or inhibition of gene expression. The methyl radical (CH_3_) is present mainly in cytosines that precede guanines (CpG dinucleotides), which are prevalent in gene promoter regions, and acts by inhibiting the binding of transcription factors to DNA. Changes in this profile are associated with changes in gene expression, which may be associated with diseases.^14^

Little is known about the DNA methylation profile in oncopediatric populations with oral mucositis. A study evaluating the effect of chemotherapy with MTX^®^ in oncopediatric children detected changes in the global methylation profile in blood cells. However, no association was found between this profile and mucositis, indicating that global methylation is a marker of exposure to chemotherapy but not inflammation.^15^ Another study evaluated the methylation profile in oral cells in the gene encoding the vitamin D receptor (*VDR*) and found no association with inflammation or chemotherapy.^16^

Based on the existing gaps regarding the DNA methylation profile in the context of chemoinduced oral mucositis and the importance of genes involved in oxidative and inflammatory stress mechanisms in the pathobiology of this condition, our objective was to investigate the DNA methylation profile in the *CAT, SOD3, IL6* and *TNF-α* genes in the oral mucosa of oncopediatric patients treated with MTX^®^ in an attempt to identify markers of oral mucositis or exposure to chemotherapy.

## Methodology

### Study design and research ethics

This was a cross-sectional observational study. The population comprised children and adolescents aged between 5 and 19 years of both sexes, including cancer patients and healthy patients recruited between July 2018 and April 2022. Oncologic children and adolescents were recruited at the reference hospital for cancer treatment in Paraíba, the Hospital Napoleão Laureano (João Pessoa, PB). According to the inclusion criteria, we selected patients diagnosed with leukemias or lymphomas and treated with a chemotherapy protocol involving MTX^®^. Patients were excluded if they had cognitive or motor alterations that made collection procedures difficult, a compromised or isolated health status with restricted contact (making it impossible to carry out the collection), or any other oral inflammation other than oral mucositis; or if they were being treated with a combination of chemotherapy and radiotherapy. Healthy patients were recruited from a Private Pediatric Dental Clinic (João Pessoa, PB). This group included patients without a diagnosis of neoplasms and without alterations in the oral mucosa. Demographic and oral health data of the patients were collected from medical and dental records and transcribed. All procedures complied with the 1964 Declaration of Helsinki and its subsequent amendments to comparable ethical standards. This research was approved by the Research Ethics Committee of the Federal University of Paraíba (UFPB) (Opinion No. 4,878,034).

### Assessment of oral condition

The assessment of the patients’ oral condition was performed by previously calibrated subjects (Kappa = 0.87) using the modified Oral Assessment Guide (OAG), an instrument that measures changes in the oral mucosa of pediatric patients due to chemotherapy. The instrument’s score ranges from 1 to 3, where 1 indicates normal mucosa, 2 indicates mild and/or moderate alterations, and 3 indicates severe complications.^17^ Eight items were evaluated: voice, swallowing, lips, tongue, saliva, labial mucosa/palate, labial mucosa, and gingiva. In accordance with hospital policy, oncopediatric patients receive preventive care from the oral health team with preventive photobiomodulation and guidance on biofilm control through oral hygiene methods. In situations where mechanical control of biofilm is impossible, a 0.12% chlorhexidine solution is prescribed. In addition to these strategies, oral preparation interventions are performed to remove active foci of infection, as well as removal of devices that may promote biofilm accumulation, such as orthodontic appliances.

### Sample selection and sample calculation

The selection of cancer patients for the collection of biological samples occurred in the first 60 days of treatment, based on the state of the disease in this period (individuals without oral mucositis and individuals with oral mucositis or who had recovered from mucositis). Patients who did not develop mucositis were followed up until the last session to ensure this condition. This period was chosen because mucositis usually appears in the first weeks of treatment, as previously addressed in a methylation study.^1,16^

The study population (n = 85) was allocated into four groups: 1- *Healthy* (n = 21): individuals without cancer; 2 – *Cancer*: (n = 16): oncopediatric patients without oral mucositis; 3 – *Cancer and mucositis* (n = 17): oncopediatric patients with oral mucositis at the time of sample collection; 4 – *Cancer who had recovered from mucositis* (n = 31): oncopediatric patients without oral mucositis at the time of sample collection, but who had already presented mucositis during chemotherapy.

Sample size calculation was performed on the website Cálculo Amostral – USP Statistics (http://estatistica.bauru.usp.br/calculoamostral/index.php), using the difference parameters between two variables. The following were considered: Pearson’s correlation coefficient r=0.3 (average coefficient, absence of previous studies); α error of 5% and β error of 80%. The calculation resulted in an estimated n of 17 patients per group.

### Methylation analysis in CAT, SOD3, IL6, TNF-*α* genes in oral mucosa

Oral mucosal cells were obtained by mouth rinsing with 5 mL of sterile 3% dextrose solution, followed by DNA extraction with 8 M ammonium acetate and 1 mM EDTA, as previously described.^18^ The quantification and purity of the DNA was measured in a spectrophotometer with the DNA OD ratio of 260/280. Values above 1.8 were considered pure (NanoDrop^®^ 2000). The samples were kept at -20°C until methylation analyses. Methylation at specific sites was performed using methylation-specific PCR (MSP), which requires treatment of DNA with sodium bisulfite to transform unmethylated cytosines into uracil in order to differentiate methylated and unmethylated sites. To perform this technique, 1,000 ng of previously purified DNA was treated with the EZ Methylation Gold Kit (Zymo Research) according to the manufacturer’s instructions. Differences in DNA sequences after bisulfite treatment were detected by amplification with specific primers for methylated and unmethylated sequences as previously described, with modifications in PCR annealing conditions when necessary (Table 1).^19-22^ Treated DNA was amplified in 20-μL reactions containing 10 μL of GoTaq^®^ G2 Hot Start Green Master Mix (Promega Corporation), forward and reverse primers, 50 ng of bisulfite-transformed DNA, and nuclease-free water. After amplification, methylation profiles were visualized by vertical electrophoresis of 10 µL of amplified DNA on 6% polyacrylamide gels, followed by silver nitrate staining. The specificity of the methylated and unmethylated reactions was ensured by the use of fully methylated (Universal Methylated Human DNA Standard, Zymo Research) and fully unmethylated DNA (EpiTect Control DNA, Qiagen), bisulfite-transformed as mentioned above, and PCR was performed. Profiles were categorized as methylated (also known as hypermethylated), with amplification only in the methylated condition, unmethylated (also known as hypomethylated), with amplification in the unmethylated condition, and partially methylated, when amplification was observed in both conditions.

**Table 1:** Methylation Specific PCR (MSP) conditions for DNA methylation analysis.

Gene/CPG Site	Primers	Primer/Volume	Anneling (°C)/Time	Cicles	Product (BP)
CAT 112, 118, 372					
M	F: GTAGACGTATATTCGTTGTCGTTATTC	10µM/1 µL	55°/40sec	35	255
	R: AAAAATCTCATTACCAAACACTTCG				
U	F: AGATGTATATTTGTTGTTGTTATTTGT	10µM/1 µL	59°/40sec	35	261
	R: AAAATCTCATTACCAAACACTTCAAA				
*SOD3 -173, -35*					
M	F: GTAGACGTATATTCGTTGTCGTTATTC	10µM/0,8 µL	60°/40sec	40	138
	R: AAAAATCTCATTACCAAACACTTCG				
U	F: AGATGTATATTTGTTGTTGTTATTTGT	10µM/0,8 µL	63°/ 60sec	40	137
	R: AAAATCTCATTACCAAACACTTCAAA				
IL6 -628, -610, -574, -491					
M	F: GTAGACGTATATTCGTTGTCGTTATTC	10µM/1 µL	60°/60sec	35	104
	R: AAAAATCTCATTACCAAACACTTCG				
U	F: AGATGTATATTTGTTGTTGTTATTTGT	10µM/1 µL	61°/60sec	35	104
	R: AAAATCTCATTACCAAACACTTCAAA				
TNF-α -245,-239					
M	F: GTAGACGTATATTCGTTGTCGTTATTC	17µM/0,7 µL			120
	R: AAAAATCTCATTACCAAACACTTCG		61°/40sec	38	
U	F: AGATGTATATTTGTTGTTGTTATTTGT	17µM/0,7 µL	61°/40sec	38	120
	R: AAAATCTCATTACCAAACACTTCAAA				

M: methylated; U:unmethylated; F: foward; R: reverse; bp: base pair; µM: micro molar; µL: microliter; sec: seconds; °C= Celsius degrees

### Statistical analysis

Data were categorized and organized in a database. Data normality was determined by the Kolmogorov-Smirnov test. The relationships between the variables were measured using the Chi-square test and Fisher’s exact test, followed by the Dwass-Steel-Critchlow-Fligner (DSCF) test for multiple comparisons, adopting α<0.05. The Jamovi 2.3.12 software (Stats Open Now, Australia) was used.

## Results

### Demographic and clinical data

A total of 85 individuals participated in the study, most of them female (n = 46; 54.1%), with a mean age of 11.1 (± 4.3) years. Regarding oncopediatric patients (n = 64), the majority was diagnosed with acute lymphoblastic leukemia (n = 48; 75%) and the others were diagnosed with other hematological neoplasms (n = 16; 25%). In addition, the majority of oncopediatric patients developed mucositis during chemotherapy treatment (n = 48; 75%). Of these, 64.5% had already recovered at the time of collection of biological material (n = 31) and 35.4% had inflammation during collection (n = 17) (Table 2).

**Table 2:** Demographic and clinical data of the study population.

Variable	Groups			

	Healthy (n = 21)	Cancer (n = 16)	Cancer and mucositis (n = 17)	Cancer who recovered from mucositis (n= 31)
Sex n (%)				
Girls	14 (66.7)	11 (68.8)	07 (41.2)	14 (45.2)
Boys	07 (33.3)	05 (31.2)	10 (58.8)	17 (54.8)
Age – average (±SD)	10.19 (± 3.26)	10.06 (± 3.64)	10.00 (± 4.33)	12.71 (± 4.73)
Underlying disease n (%)				
ALL	0	11 (68.7)	12 (70.6)	25 (80.6)
AML	0	02 (12.4)	02 (11.8)	02 (6.5)
APL	0	01 (6.3)	0	02 (6.5)
CML	0	01 (6.3)	0	01 (3.2)
HL	0	01 (6.3)	0	0
NHL	0	0	03 (17.6)	01 (3.2)
No cancer	21 (100.0)	0	0	0

ALL: acute lymphoblastic leukemia; AML: acute myeloid leukemia; APL: acute promyelocytic leukemia; CML: chronic myeloid leukemia; HL: Hodgkin’s lymphoma; NHL: non-Hodgkin lymphoma.

### DNA methylation data

**Figure 1:**
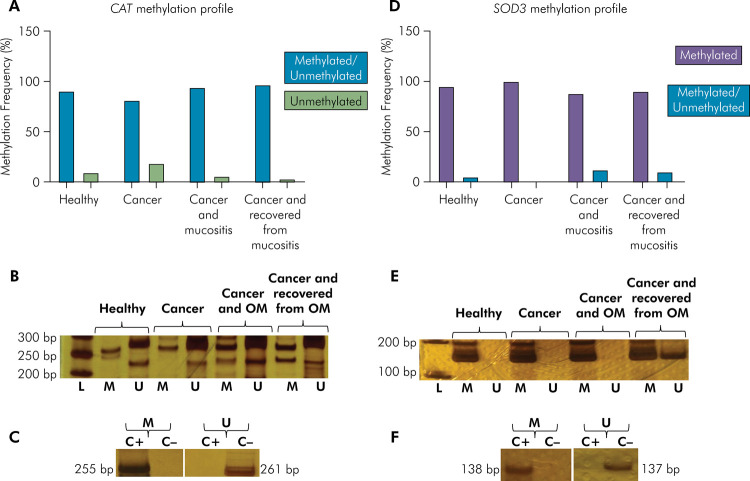
*CAT* and *SOD3* methylation profile in oral cells of the study population (n=85). (A) Methylation frequency for *CAT.* (B) Representative bands showing *CAT* MSP reactions. (C) Representative bands showing the specificity of *CAT* MSP reactions. (D) Methylation frequency for *SOD3.* (E) Representative bands showing *SOD3* MSP reactions. (F) Representative bands showing the specificity of *SOD3* MSP reactions.

For *CAT*, there was a prevalence of the partially methylated (methylated and unmethylated) profile in the four groups (> 80%), with no one with fully methylated profile and a small percentage of individuals had a unmethylated profile. No significant difference was identified between groups (p = 0.302; Fisher’s exact test). For *SOD3*, a higher frequency of the fully methylated profile was observed in all groups (> 88%), with no unmethylated profiles and a few individuals with a partially methylated profile. Again, no significant difference was detected between groups (p = 0.622; Fisher’s exact test) (Figure 1).

L: DNA ladder; bp: base pair; M: methylated; U: unmethylated; C+: fully methylated DNA; C-: fully unmethylated DNA; OM: oral mucositis. Note: methylated and unmethylated profiles are also known as partially methylated. Methylated profile is also known as hypermethylated. Unmethylated profile is also known as hypomethylated.

**Figure 2:**
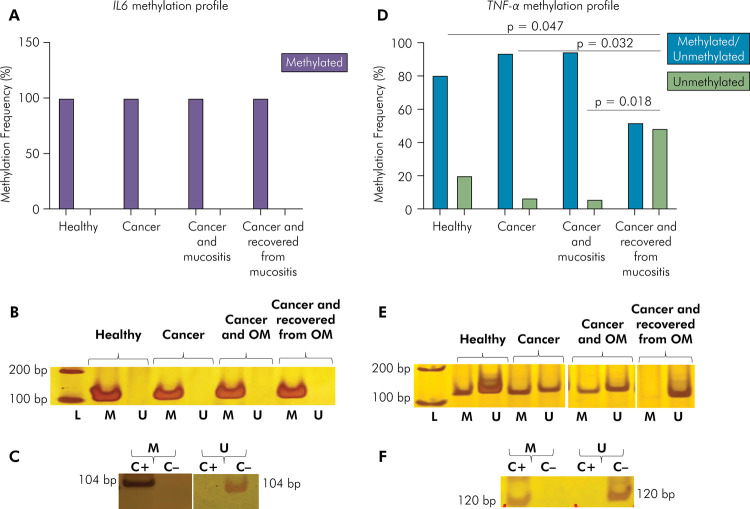
*IL6* and *TNF-α* methylation profile in oral cells of the study population (n=85). (A) Methylation frequency for *IL6.* (B) Representative bands showing *IL6* MSP reactions. (C) Representative bands showing the specificity of *IL6* MSP reactions. (D) Methylation frequency for *TNF-α.* (E) Representative bands showing *TNF-α* MSP reactions. (F) Representative bands showing the specificity of *TNF-α* MSP reactions.

For *IL6*, the fully methylated profile was found in all investigated individuals (100%). A higher frequency of the unmethylated profile (48%) of *TNF-α* was found in oncopediatric patients who recovered from mucositis at the time of sample collection (p = 0.002; Fisher’s exact test), than in healthy patients (20%) (p = 0.047; Dwass-Steel-Critchlow-Fligner multiple comparisons test) and oncopediatric patients with mucositis (6%) (p = 0.018; Dwass-Steel-Critchlow-Fligner multiple comparisons test) and without mucositis (7%) (p = 0.032; Dwass-Steel-Critchlow-Fligner multiple comparisons test) (Figure 2).

L: DNA ladder; bp: base pair; M: methylated; U: unmethylated; C+: fully methylated DNA; C-: fully unmethylated DNA; OM: oral mucositis. Note: methylated and unmethylated profiles are also known as partially methylated. Methylated profile is also known as hypermethylated. Unmethylated profile is also known as hypomethylated.

## Discussion

The pathobiology of chemotherapy-induced oral mucositis involves oxidative stress and inflammation.^4,5^ In addition, the DNA methylation profile has been identified as a marker of oral inflammation and exposure to chemotherapy.^15,23^ In this context, our objective was to evaluate the methylation profile of genes involved in of oxidative stress and inflammation mechanisms in oncopediatric patients with chemotherapy-induced oral mucositis.

The enzymes catalase and superoxide dismutase 3 are important antioxidant agents that are located in the peroxisome and in the extracellular environment, respectively. During oxidative stress, cells respond to ROS (reactive oxygen species) with SOD, which is responsible for destroying superoxide radicals (O^2^), converting these radicals to molecular oxygen (O_2_) and hydrogen peroxide (H_2_O_2_). Catalase, in turn, converts H_2_O_2_ into O_2_ and H_2_O.^6^ The *CAT* and *SOD3* genes, which encode catalase and superoxide dismutase 3, respectively, are expressed in the oral mucosa,^24^ and since they can be epigenetically regulated and are associated with diseases,^11,12^ they are interesting targets for the study of chemo-induced oral mucositis.

Regarding the *CAT* methylation profile, our data showed a higher frequency of the partially methylated profile in all groups, with no association with inflammation or exposure to chemotherapy. The CpG sites investigated in the present study, 112, 118 and 372, were previously analyzed by Ding et al.,^19^ who observed a partially methylated profile in hepatocellular carcinoma. The methylation profile at the CpG −47 site of *CAT* was studied in oral cells of adult subjects and an increase in methylation frequency was a trend for the group with periodontitis.^23^ Comparing the data of Ding et al.^19^ and Coêlho et al.,^23^ it is clear that the frequency of *CAT* methylation, even considering different CpG sites, tended to increase in tumor and inflammatory conditions. Methylation may be associated with a decrease in gene expression and, consequently, a lower level of catalase, causing accumulation of H_2_O_2_, which leads to tissue damage. However, in the present study, we did not observe an increase in methylation tendency in the mucositis group, with the majority of individuals having the partially methylated profile. Thus, the data suggest that the partially methylated profile at CpG sites 112, 118 and 372 of *CAT* is a common profile for cells in the oral mucosa of children and adolescents and has no association with inflammation or chemotherapy exposure.

For *SOD3*, the fully methylated profile was the most frequent in the study population and no significant differences were found between groups. The *SOD3* CpG sites considered in the present study (−173 and −35 sites) were previously analyzed by Kamiya et al.,^20^ who also observed a fully methylated profile in human monocytes of the THP-1 type. The authors treated the cells with 12-O-tetra decanoylphorbol-13-acetate (TPA), and also found no alteration in the DNA methylation profile, but detected *SOD3* expression in THP-1 cells, indicating that *SOD3* expression can be induced but is not related to the change in the methylation profile at these CpG sites in these cells. Griess et al.^12^ studied six CpG sites of *SOD3* promoter, including −35, and found higher levels of methylation in breast neoplastic cells than in healthy breast cells. In addition, these data were correlated with expression level and histological condition. Comparing the studies by Kamiya et al.^20^ and Griess et al.,^12^ we note that the methylated profile is common for neoplastic cells, contrary to our findings in which the methylated profile was frequent in non-tumor oral cells. It is known that the methylation profile is tissue-, site-, and age-specific^13,14,25^and to the best of our knowledge there is no other study in the literature showing the methylation profile of *SOD3* in oral cells for comparison. Thus, our findings suggest that the methylated profile at the CpG −173 and −35 sites of *SOD3* is a common profile for oral epithelial cells in children and adolescents.

In addition to oxidative stress, another important factor in the pathobiology of mucositis is the pro-inflammatory markers interleukin-6 and tumor necrosis factor-alpha, which seem to be mediated by oxidative stress.^5^ The *IL6* and *TNF-α* genes that encode interleukin-6 and tumor necrosis factor-alpha, respectively, are expressed by oral mucosa cells,^26^ can be epigenetically regulated, and are associated with diseases.^9,10^

For *IL6*, the fully methylated profile at CpG sites −628, −610, −574, and −491 was found in all subjects with no association with inflammation or chemotherapy exposure. The same CpG sites were previously studied by Stefani et al.^21^ in adult gingival tissue and the partially methylated profile was the most frequently observed in individuals with and without periodontitis, thus indicating that the methylation profile at these sites is not associated with inflammation, as in our work. The difference in methylation profiles between the study by Stefani et al. (partially methylated) and the present study (fully methylated) may be due to the use of different cell types and the age of the population studied.

In blood cells from adults with and without periodontitis, high levels of methylation at the −491 site and unmethylation at the −610 site were observed and no association with inflammation was detected.^9^ In blood cells from adults with and without autoimmune disease, moderate methylation was observed at site −491 and a very low level of methylation was observed at −610.^27^ The data from the aforementioned studies and the present study indicate that the level of methylation varies at sites −491 and −610 depending on the tissue and age of the population, but all studies showed no association with inflammation. Thus, our data show that the fully methylated (hypermethylated) profile at CpG sites −628, −610, −574, and −491 is a common feature of oral cells from children and adolescents and that there is no association with inflammation and exposure to chemotherapy.

For *TNF-α*, the unmethylated (hypomethylated) profile at CpG sites −245 and −239 was more frequent in patients who had oral mucositis during treatment but recovered and were not inflamed at the time of collection. These results suggest that inflammation can modify the *TNF-α* methylation profile at the analyzed sites in the long term. That is, during the acute phase of oral mucositis, the process of DNA demethylation can begin, which in turn increases over time until the unmethylated profile is established. In fact, Zhang et al.^10^ evaluated the methylation profile of *TNF-α* in the course of periodontal disease. The authors did not observe a change in the methylation profile during the acute phase (gingivitis), but found that this profile changed over time, indicating that epigenetic alterations might be locally sustained for some mediators even after inflammation subsides.

The same CpG sites studied in the present work were evaluated by Gomes et al.,^22^ who observed hypomethylation in blood cells of individuals infected with the dengue virus. This profile was associated with increased levels of *TNF-α* mRNA, indicating an association between methylation profile at CpG sites −245 and −239 and gene expression. Another study found hypomethylation at CpG sites −55, −89, −132, −189, −237, and −298 in cartilage cells from individuals with osteoarthritis,^28^ which was also associated with increased levels of both mRNA and cytokine. Taken together, these data show that the CpG sites of the *TNF-α* promoter region are likely to be modulated by inflammation, and are associated with gene expression, where hypomethylation is associated with increased expression of *TNF-α*, a pro-inflammatory cytokine that is important in phase 2 oral mucositis.^4,5^

The demethylation process (the hypothesis of the present study for patients who recovered from mucositis) may be associated with a decrease in the expression of DNA methyltransferases (enzymes that add the methyl radical to DNA). A study with inflamed dental pulp cells showed a decrease in the expression of DNMT1 and an increase in the expression of inflammatory cytokines. In addition, increased cytokine expression was associated with a hypomethylated profile in the genes that encode these cytokines.^29^ Interestingly, we have previously observed that in the group of patients who recovered from mucositis, there was an increased frequency of *DNMT1* methylation,^30^ and here we observed *TNF-α* hypomethylation. It is possible that the decrease in *DNMT1* expression caused by hypermethylation leads to *TNF-α* hypomethylation and its consequent increase in expression.

In addition, a study has shown that *TNF-α* is important for fracture healing and excessive or insufficient levels may impede the healing process.^31^ Also, blocking TNF-α with infliximab, a monoclonal antibody to human TNF-α, has been shown to reduce acute inflammatory response during the development of traumatic oral ulcers, but may delay wound healing in mucosal injuries.^32^ Therefore, this cytokine has been shown to play a role in the development of mucositis (phase 2 and 3), as previously demonstrated in the literature, but it may also be important in the healing process (phase 5).

The limitations of this study include the fact that it was not possible to separate individuals by disease severity for statistical analysis and to assess the association between severity and methylation profile due to the small sample size. In addition, a quantitative methylation analysis would be needed to show whether changes in the methylation profile of *TNF-α* at the time of mucositis can indeed be detected, which is our hypothesis. Despite these limitations, our unpublished data contribute to the understanding of the molecular mechanisms involved in chemo-induced oral mucositis and indicate that the *TNF-α* gene plays an important role in this process. We also raise interesting questions: How long does the hypomethylated *TNF-α* profile last after mucositis resolves? Is this profile associated with *TNF-α* expression? These questions can be answered with long-term follow-up of patients, including studies before and after chemotherapy and quantification of mRNA and *TNF-α* levels.

The first study addressing the methylation profile of oncopediatric patients treated with MTX^®^ showed a change in the global methylation profile in blood cells after treatment, but found no association with oral mucositis.^15^ The second study investigated site-specific methylation in the gene that encodes the vitamin D receptor (*VDR*) in oral cells and found no association with treatment or mucositis.^16^ The third study demonstrated hypermethylation of the *DNMT1* gene in patients who recovered from mucositis.^30^ Here we found a higher frequency of hypomethylation of the *TNF-α* gene in oral cells of patients who recovered from mucositis. Although we did not find differences in the methylation profile of all the genes studied, it is possible that other CpG sites in these genes could be modulated by inflammation or chemotherapy, since studies have shown that oral inflammation as well as the effect of chemical agents might be associated with site-specific changes.^9,33^ In addition to DNA methylation being a potential biomarker of inflammation, data from these studies have great potential to serve as both diagnostic and therapeutic tools.

## Conclusion

Hypomethylation of *TNF-α* is associated with recovery of oral mucositis due to chemotherapy in oncopediatric patients. For *CAT*, SOD, and *IL6*, there was no change in the methylation profile during mucositis or exposure to MTX^®^.
